# S-Protected Thiolated Chitosan versus Thiolated Chitosan as Cell Adhesive Biomaterials for Tissue Engineering

**DOI:** 10.1021/acsami.3c09337

**Published:** 2023-08-18

**Authors:** Bao Le-Vinh, Christian Steinbring, Nguyet-Minh Nguyen Le, Barbara Matuszczak, Andreas Bernkop-Schnürch

**Affiliations:** † Department of Pharmaceutical Technology, Institute of Pharmacy, University of Innsbruck, Innrain 80/82, 6020 Innsbruck, Austria; ‡ Department of Industrial Pharmacy, Faculty of Pharmacy, University of Medicine and Pharmacy at Ho Chi Minh city, 700000 Ho Chi Minh City, Vietnam; § Department of Pharmaceutical Chemistry, Institute of Pharmacy, University of Innsbruck, Innrain 80-82, 6020 Innsbruck, Austria

**Keywords:** disulfide, thiolated polymers, thiomers, scaffold, cryogel, cell adhesion

## Abstract

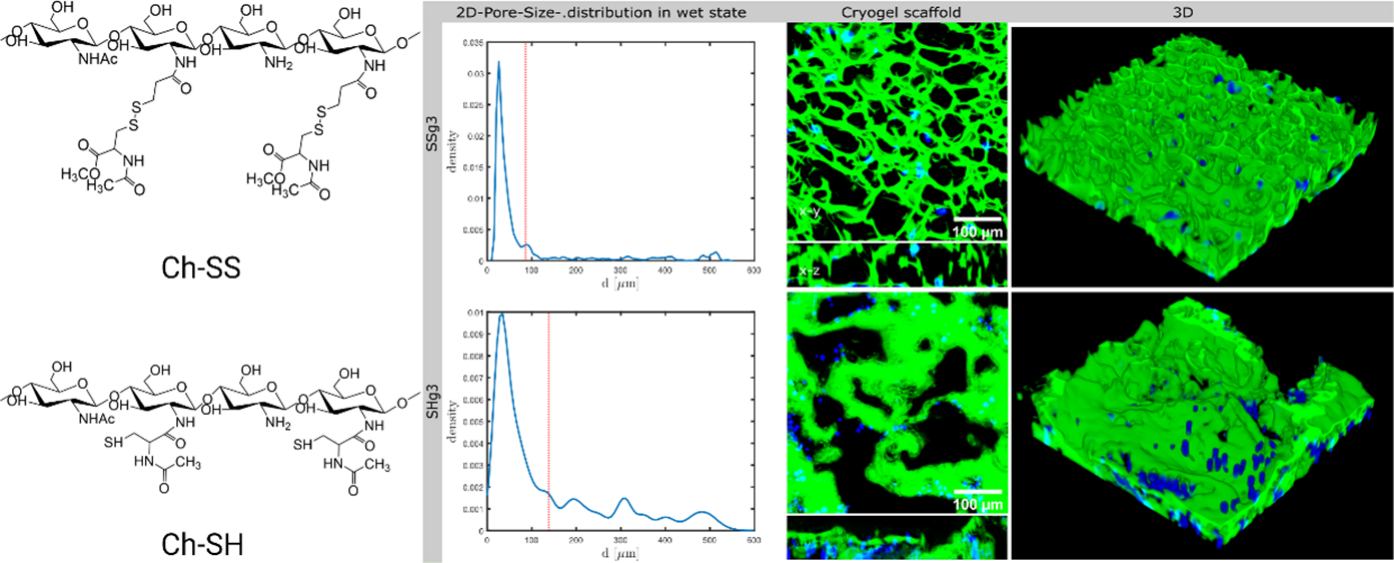

Chitosan (Ch) and different Ch derivatives have been applied in tissue engineering (TE) because of their biocompatibility, favored mechanical properties, and cost-effectiveness. Most of them, however, lack cell adhesive properties that are crucial for TE. In this study, we aimed to design an S-protected thiolated Ch derivative exhibiting high cell adhesive properties serving as a scaffold for TE. 3-((2-Acetamido-3-methoxy-3-oxopropyl)dithio) propanoic acid was covalently attached to Ch via a carbodiimide-mediated reaction. Low-, medium-, and high-modified Chs (Ch-SS-1, Ch-SS-2, and Ch-SS-3) with 54, 107 and 140 μmol of ligand per gram of polymer, respectively, were tested. In parallel, three thiolated Chs, namely Ch-SH-1, Ch-SH-2, and Ch-SH-3, were prepared by conjugating *N*-acetyl cysteine to Ch at the same degree of modification to compare the effectiveness of disulfide versus thiol modification on cell adhesion. Ch-SS-1 showed better cell adhesion capability than Ch-SS-2 and Ch-SS-3. This can be explained by the more lipophilic surfaces of Ch-SS as a higher modification was made. Although Ch-SH-1, Ch-SH-2, and Ch-SH-3 were shown to be good substrates for cell adhesion, growth, and proliferation, Ch-SS polymers were superior to Ch-SH polymers in the formation of 3D cell cultures. Cryogels structured by Ch-SS-1 (SSg) resulted in homogeneous scaffolds with tunable pore size and mechanical properties by changing the mass ratio between Ch-SS-1 and heparin used as a cross-linker. SSg scaffolds possessing interconnected microporous structures showed good cell migration, adhesion, and proliferation. Therefore, Ch-SS can be used to construct tunable cryogel scaffolds that are suitable for 3D cell culture and TE.

## 1 Introduction

Chitosan (Ch), a naturally derived polysaccharide, consists of D-glucosamine and *N*-acetyl-D-glucosamine subunits connected by β-1,4 glycosidic linkages. It has a similar structure to glycosaminoglycans (GAGs)––the major components of the extracellular matrix (ECM). It has been employed as a scaffolding material in tissue engineering (TE) for the construction of a wide variety of TE platforms, especially in wound healing, bone, cartilage, nerves, liver, blood vessels, and muscle TE^1−5^ because of its intrinsic antimicrobial properties and ability to accelerate healing by increasing the rate of infiltration of fibroblasts at the wound site and consequentially collagen production.^6^ Furthermore, Ch is less immunogenic, biocompatible, controllably degraded in vivo with nontoxic degradants,^7,8^ and unlike other natural polymers used in TE, cost-effective and available in large quantities. Ch itself, however, provides limited cell adhesion^9−11^ although cell–substrate adhesion is of utmost importance for cell growth, proliferation, and differentiation.^3,12,13^

In order to address this shortcoming, it was the aim of this study to design a Ch derivative, which has high cell adhesive properties, as a scaffolding material for TE. It is well-known that the cell surface and proteins involved in the cell–substrate adhesion process express or contain thiol and disulfide groups^14−18^ and cells can adhere to substrates via disulfide bonds––the most important bridging structure designed by nature. We synthesized cell adhesive Ch derivatives by introducing disulfide substructures to Ch polymer backbones. As the thiol/disulfide exchange reactions between scaffolding polymers and cell surface/cell adhesion proteins are mainly responsible for the formation of disulfide bonds, both free thiols and disulfides attached to Ch can form new disulfide bonds with cellular surfaces. Since their introduction as scaffold materials for TE at the 4th Central European Symposium on Pharmaceutical Technology in 2001,^19^ various thiolated Chs have been synthesized and showed promising results for this application.^20^ However, in most cases, these thiolated Chs were used in combination with other cross-linkers^21,22^ or polymers to generate hydrogels^23^ or polyelectrolyte multilayers,^24^ and the scaffolds were, in many cases, further functionalized with cell adhesive peptides like RGD (arginine-glycine-aspartic acid)-containing peptide,^23^ BMP2-derived peptide,^21^ or Histatin-1.^22^ Consequently, less is known about the cell adhesive properties of thiolated Chs per se, and in particular, cell adhesive properties of S-protected thiolated Ch in both 2D and 3D forms are still unknown.

In this study, the disulfide-bearing ligand, 3-((2-acetamido-3-methoxy-3-oxopropyl)dithio) propanoic acid was synthesized and attached to Ch at different degrees of conjugation mediated by a carbodiimide. These S-protected thiolated Chs were characterized and evaluated for their cell adhesive properties on polymer membranes in comparison with the well-studied thiolated Ch, *N*-acetyl cysteine Ch.^25^ Furthermore, cryogels from S-protected thiolated Chs and thiolated Chs using heparin as a cross-linker were prepared. Their swelling and rheological properties as well as the migration, adhesion, and proliferation of cells in 3D structures were evaluated.

## 2 Materials and Methods

### 2.1 Materials

Chitosan 85/100 (degree of deacetylation, 82.6–87.5%; viscosity of 1% solution in 1% acetic acid, 71–150 mPa s; and average molecular weight (*M*_w_), 220 kDa) was purchased from Heppe Medical Chitosan GmbH, Germany. Heparin sodium salt of *M*_w_ 12–15 kDa and 3-(2-pyridyldithio) propionic acid (PDP) were purchased from Biosynth Carbosynth, UK. N-acetyl L-cysteine (Nac), *N*-acetyl L-cysteine methyl ester (NacME), N-ethyl-N′-(3-dimethylaminopropyl)-carbodiimide hydrochloride (EDC), N-hydroxysuccinimide (NHS), N-hydroxysulfosuccinimide (sNHS), 5,5′-dithiobis(2-nitrobenzoic acid) (DTNB), Supelco silica gel high-purity grade 60 Å/230–400 mesh, Dulbecco’s modified Eagle’s medium (DMEM), MEM Eagle powder, phosphate-buffered saline (PBS) Dulbecco without Ca^2+^ and Mg^2+^, (4-(2-hydroxyethyl)-1-piperazineethanesulfonic acid) (HEPES), Triton X100, and methylthiazolyldiphenyl-tetrazolium bromide (MTT) were purchased from Sigma-Aldrich, Austria. Fetal bovine serum (FBS) superior was obtained from Biochrom GmbH, Germany. Penicillin–streptomycin solution (PS) containing 10,000 U/mL penicillin and 10 mg/mL streptomycin was obtained from Pan Biotech, Germany. All other chemicals were of analytical grades.

Human colon adenocarcinoma HT29 cells (ECACC 91072201) were obtained from the European Collection of Authenticated Cell Cultures, UK. Mouse embryonic fibroblast cells NIH-3T3 (3T3) were a gift from Universitätsklinik für Dermatologie, Venerologie und Allergologie Innsbruck, Austria. Rat chondrocytes, isolated and cultured as described in the Supporting Information, passage number 2–5, were used for experiments.

Hank’s balanced salt solution (HBSS) containing 8 g/L NaCl, 0.185 g/L CaCl_2_, 0.4 g KCl, 0.1 g/L MgSO_4_, 0.06 g KH_2_PO_4_, 0.05 g/L Na_2_HPO_4_, 0.35 g NaHCO_3_, 1 g/L d-glucose, and 10 mM HEPES was prepared and adjusted to pH 7.4. HBSS was sterilized by filtration through a 0.2 μm membrane and stored at 4 °C. Ellman’s reagent solution was prepared by dissolving 3 mg of DTNB in 10 mL of 0.1 M sodium phosphate buffer pH 8.

### 2.2 Synthesis of the Disulfide-Bearing Ligand

**Figure 1:**
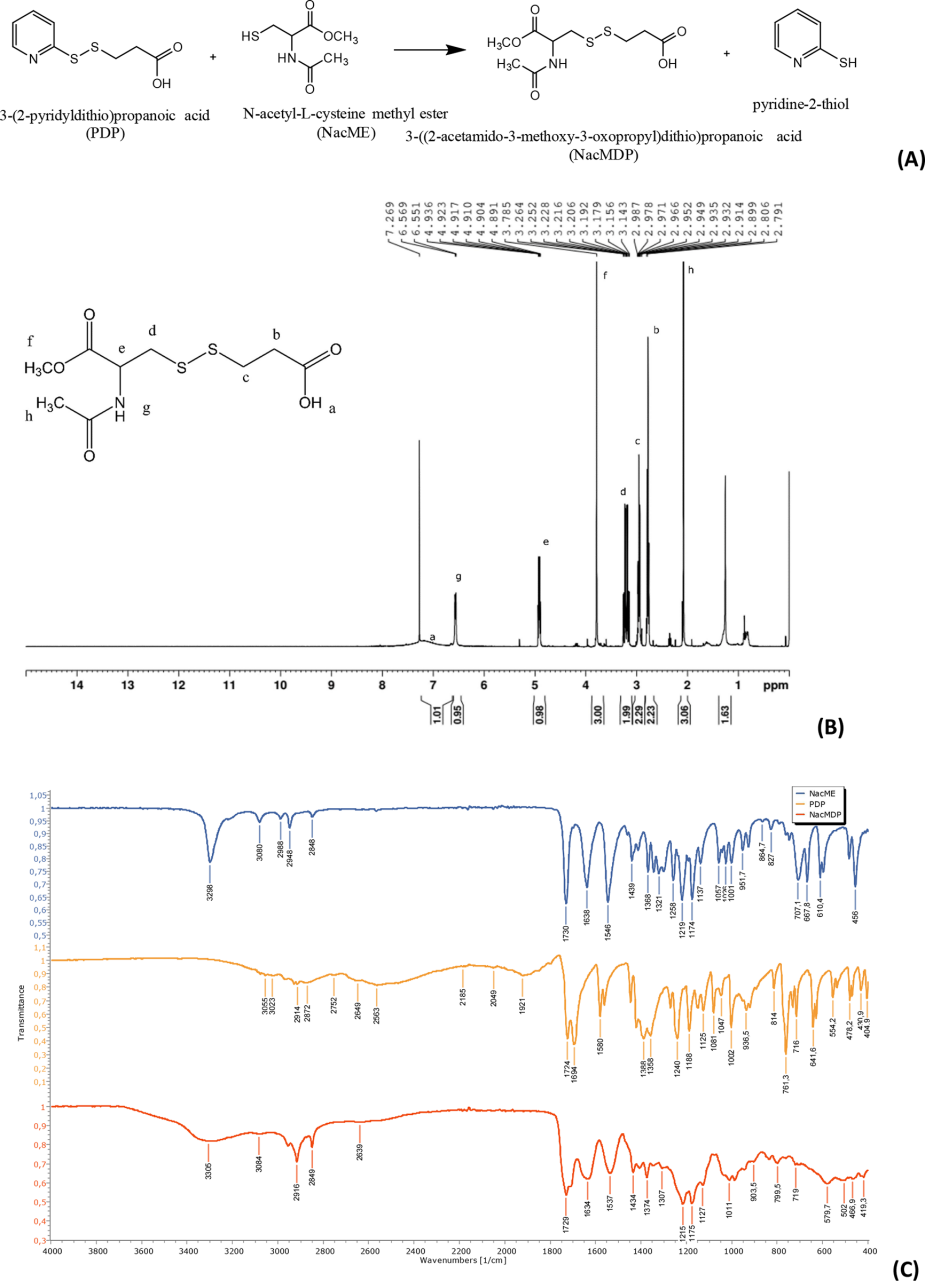
(A) Synthesis of the disulfide-bearing ligand 3-((2-acetamido-3-methoxy-3-oxopropyl)dithio) propanoic acid (NacMDP) by thiol-disulfide exchange reaction between PDP and NacME. (B) ^1^H NMR of the disulfide-bearing ligand NacMDP recorded in CDCl_3_. ^1^H NMR (CDCl_3_) NacMDP: 2.08 (s, 3H, CH_3_), 2.77 (t, *J* = 6.8 Hz, 2H, CH_2_), 2.97–2.93 (m, 2H, CH_2_), 3.14–3.26 (m, 2H, CH_2_), 3.79 (s, 3H, OCH_3_), 4.89–4.94 (m, 1H, CH), 6.56 (d, *J* = 7.2 Hz, 1H, NH), 6.8–7.5 (s br, 1H, COOH) ppm. (C) FTIR spectra (from top to bottom) of N-acetyl L-cysteine methyl ester (NacME), 3-(2-pyridyldithio) propionic acid (PDP), and the ligand product 3-((2-acetamido-3-methoxy-3-oxopropyl)dithio) propanoic acid (NacMDP).

N-acetyl L-cysteine methyl ester (NacME) was reacted with 3-(2-pyridyldithio) propionic acid (PDP), generating the disulfide-bearing ligand 3-((2-acetamido-3-methoxy-3-oxopropyl)dithio) propanoic acid (NacMDP) and pyridine-2-thiol as the leaving group (Figure 1A). Briefly, 100 mg (0.464 mmol) of PDP was dissolved in 5 mL of ethanol and 0.2 mL of glacial acetic acid, whereas 41.2 mg of NacME (0.232 mmol) was dissolved in 2 mL of ethanol. The NacME solution was added dropwise to the PDP solution under vigorous stirring in a round-bottom flask at room temperature. Subsequently, the flask was purged with nitrogen and sealed with a Teflon cap. The reaction product was monitored by normal-phase thin-layer chromatography (TLC), with the mobile phase consisting of dichloromethane:ethanol:acetic acid (95:5:0.6). After 24 h, the solvent was removed by a rotary evaporator (Heidolph Hei-VAP value + Vacuubrand CVC 3000, Germany). 3-((2-Acetamido-3-methoxy-3-oxopropyl)dithio) propanoic acid (NacMDP) was separated by column chromatography. Accordingly, 15 g of silica gel was packed in a chromatography fritted column having an inner diameter of 2 cm and length of 30 cm by a slurry method. Crude reaction mixture was then loaded on the chromatography column and eluted with a mobile phase consisting of 95:5 dichloromethane:ethanol. After the dead volume, fractions of 2 mL were collected. Fractions that contained the target product (confirmed by TLC and further confirmed by FTIR and ^1^H NMR) were pooled and concentrated using the rotary evaporator. The residue was dissolved in a small amount of water and freeze-fried (Christ Gamma 1–16 LSC, Germany). The final product was aliquoted and stored at −20 °C for further use.

### 2.3 Synthesis of Thiolated Chitosan and S-Protected Thiolated Chitosan

**Figure 2:**
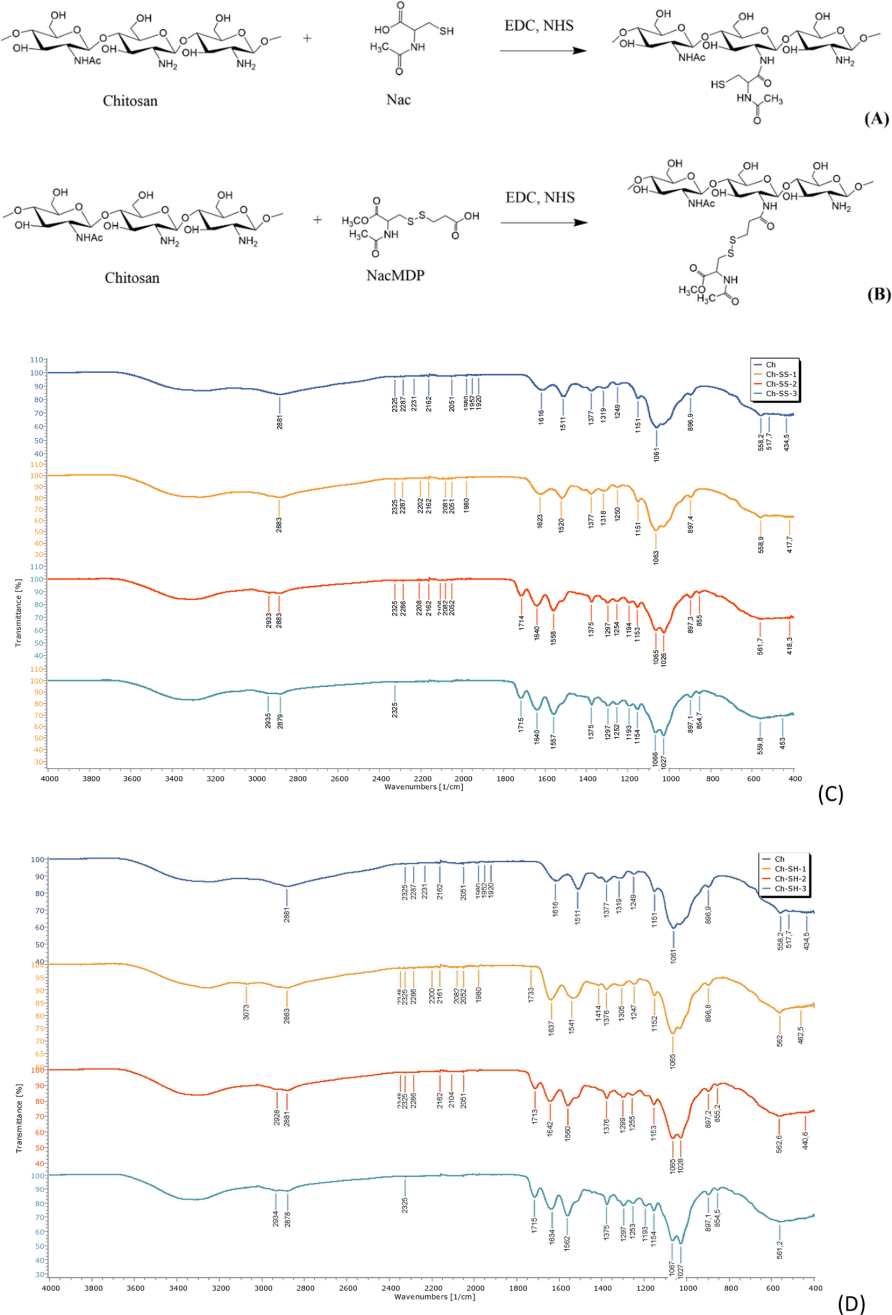
Synthesis of the thiolated chitosan––chitosan–Nac conjugate (Ch-SH) (A) and S-protected thiolated chitosan––chitosan–NacMDP conjugate (Ch-SS) (B) by the amidation of amine groups on chitosan backbones, with EDC and NHS as catalysts. (C) FTIR spectra of chitosan (Ch) and S-protected thiolated chitosans Ch-SS-1, Ch-SS-2, and Ch-SS-3 corresponding to the NacMDP:NH_2_ molar ratios of 1:10, 1:5, and 1:2, respectively. (D) FTIR spectra of chitosan (Ch) and thiolated chitosans Ch-SH-1, Ch-SH-2, and Ch-SH-3 corresponding to the Nac:NH_2_ molar ratios of 1:10, 1:5, and 1:2.

The synthesis of thiolated Ch––Ch-Nac conjugate (Ch-SH)––and S-protected thiolated Ch––Ch-NacMDP conjugate (Ch-SS)––via amidation reaction using EDC and NHS as catalysts is illustrated in Figure 2. First, 0.2 g of Ch was hydrated in 100 mL of water and dissolved by adding 2 mL of 5 M HCl. The pH of the Ch solution was then adjusted to 5 with 5 M NaOH. 1 g of Ch contained ∼4.6 mmol amine groups (−NH_2_), and the molar ratios of −COOH (of Nac and NacMDP) and −NH_2_ used were 1:10, 1:5, and 1:2. The corresponding amount of Nac or NacMDP was dissolved in 40 mL of ethanol. Tenfold molar amounts of EDC and NHS were added to the Nac or NacMDP solution to activate the carboxylic groups. The obtained solution was added slowly to the Ch solution with stirring. The reaction mixture was kept at 40 °C, and its pH was monitored and adjusted to the range of 4.5–5. After 24 h of reaction, the reaction mixture was adjusted to pH 6 and transferred to a dialysis bag (Nadir, Carl Roth, *M*_w_ cutoff, 10–20 kDa). Dialysis against deionized water was carried out for 2 days, with water changing thrice a day. A dried product was obtained by lyophilization and further washed with ethanol five times to remove the remaining EDC or NHS if any. Clean products were redissolved in water and lyophilized.

### 2.4 FTIR Analysis

FTIR spectra were recorded on a Spectrum Two FT-IR spectrometer (Perkin Elmer, UK) with an attenuated total reflectance (ATR) accessory. Spectra were obtained in the range of 4000–400 cm^–1^, at a resolution of 4 cm^–1^, and from an average of 10 scans.

### 2.5 ^1^H NMR Analysis

^1^H-NMR spectrum was recorded on a ″Mars″ 400 MHz Bruker Avance 4 Neo spectrometer in CDCl_3_. Chemical shifts are expressed in ppm downfield relative to tetramethylsilane, and the coupling constants (*J*) are reported in Hertz. Data for the ^1^H-NMR spectra are reported as follows: s = singlet, br s = broad singlet, d = doublet, t = triplet, m = multiplet.

### 2.6 Determination of Free Thiol and Disulfide Contents

Free thiol groups were determined according to a previously described method.^26^ Briefly, 0.5 mg of polymer was hydrated in 0.5 mL of deionized water in a 2 mL Eppendorf tube for 30 min. Afterward, 0.5 mL of Ellman’s reagent solution was added to each sample. The mixture was incubated at room temperature in the dark for 120 min. Samples were centrifuged for 5 min (Eppendorf MiniSpin, Germany). Subsequently, 100 μL of the supernatant was transferred to a 96-well microtiter plate. The absorbance of the sample at 410 nm was measured by a microplate reader (Tecan Spark, Austria). To calculate the concentration of free thiol in each sample, a calibration curve was established by plotting absorbance values against concentrations of a series of Nac standard solutions in the range of 0–0.3 mM.

To determine the amount of disulfide groups, we first determined the total amount of thiol after reducing the disulfide bonds in polymers by NaBH_4_ and then calculated the amount of disulfide groups by eq 1.
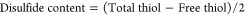
1

Accordingly, 0.5 mg of polymer was hydrated in 0.5 mL of 50 mM Tris buffer pH 7.6 in a 15 mL Falcon tube for 30 min. Afterward, 1 mL of 4% NaBH_4_ was added, and the sample was incubated at 37 °C for 120 min. Subsequently, 0.25 mL of 5 M HCl was slowly added to each sample, followed by the addition of 1 mL of 1 M sodium phosphate buffer pH 8.0. Finally, 100 μL of Ellman’s reagent solution was added, and the sample was incubated at room temperature in the dark for 90 min. The absorbance of the sample was measured in the same way, as described in the free thiol assay. All experiments were carried out in triplicate.

### 2.7 Cytotoxicity

Cytotoxicity of Ch-SH and Ch-SS was evaluated by the resazurin assay on rat chondrocytes, 3T3, and HT29 cell lines. Resazurin assay assesses cell viability via the ability of living cells to reduce the nonfluorescent resazurin to the red fluorescent resorufin. The amount of resorufin generated is proportional to the number of viable cells.

For this purpose, cells were seeded on a 24-well cell culture plate (Greiner Bio-One, Austria) at a density of 1 × 10^4^ cells/cm^2^ in DMEM containing 10% FBS and 1% PS. Cells were cultured for 5 days in a cell incubator (HeraCell 150i, ThermoScientific, Germany), adjusted at 37 °C, 95% humidity, and 5% CO_2_. The medium was changed every 2 days. To prepare the test sample, 0.5 mg of the polymer was hydrated and dispersed in 0.5 mL of culture medium in the incubator for 6 h. Thereafter, the medium in each well was replaced with the mixture of swollen polymers in the culture medium. The culture medium and 0.1% Triton X100 in the culture medium were used as negative and positive controls, respectively. All samples were prepared in at least three replicates. After 24 h of incubation, the test samples were removed, and the cell layers were washed twice with phenol red-free medium (RFM) prepared from MEM Eagle powder following manufacturer’s instructions. Subsequently, 250 μL of 44 μM resazurin in RFM was added to each well, and the plate was further incubated for 2 h. Afterward, 100 μL of the supernatant in each well was transferred to a black 96-well microtiter plate. Fluorescence intensities were measured at an excitation wavelength λ_ex_ of 540 nm and an emission wavelength λ_em_ of 590 nm using the Tecan microplate reader. Cell viability was calculated according to eq 2:
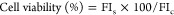
2where FI_s_ and FI_c_ are the average fluorescence intensity of the test sample and negative control, respectively.

### 2.8 Cell Attachment and Growth on Chitosan-, Ch-SH-, and Ch-SS-Coated Surfaces

To investigate the potential of thiolated Ch and S-protected Ch as biomaterials suitable for cell culture and TE, the adherence and proliferation capabilities of HT29, 3T3 cells, and rat chondrocytes on polymer-coated surfaces were evaluated. First, plastic Petri dishes (Greiner Bio-One, Austria; diameter, 35 mm; nontreated polystyrene: surface area, 9.5 cm^2^ and filling volume, 2–3 mL) were coated with Ch, Ch-SH, and Ch-SS. In brief, Ch-SH and Ch-SS were hydrated and dissolved in ethanol:water 2:8 v/v mixture to form 1% m/v pseudosolutions. In the case of Ch, 0.05 M HCl was used instead of water. The Petri dish was rinsed with ethanol. Subsequently, 200–300 μL of 1% m/v polymer solution was evenly spread on the surface of the Petri dish by a spatula and left to dry under the fume hood overnight. Polymer films on dried dishes were neutralized by immersing in 0.1 M NaOH for 30 min and then washed twice with water for 5 min each. Thereafter, water was removed, and the dishes were left to dry. The polymer-coated dishes were UV-sterilized in a laminar flow hood (LAF, Bioair Aura 200 M.A.C) for 60 min. Before cell seeding, the dishes were immersed in culture medium for 30 min to hydrate the polymer films. Cells were seeded at a density of 3 × 10^4^ cells/cm^2^ in 2 mL of culture medium. As controls, cells were plated in a six-well cell culture plate (Greiner Bio-One, Austria; surface area, 9.6 cm^2^) at the same cell density. After 4 h, the medium was withdrawn to remove unbound cells and replaced with fresh medium. Cells were cultured for 10 days, with the medium changed after every 2 days. At predetermined time points of 4 h, 2 d, 5 d, and 10 d, cell viability was assessed by resazurin assay, and the percentage of living cells in the polymer-coated Petri dish compared to that in the six-well plate was calculated according to eq 3. The resazurin assay was carried out in the same way as described in Section 2.7 with some adjustments. Accordingly, 750 μL of 44 μM resazurin in RFM was added to each dish/well after medium removal and washed with RFM, and the cells were further incubated for 3 h.
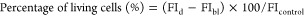
3where FI_d_ and FI_control_ are the average fluorescence intensities of the test samples from the polymer-coated dishes and six-well cell culture plate, respectively. FI_bl_ is the average value of fluorescence intensity from the corresponding polymer-coated dishes without cell seeding.

The morphology and growth of cells were observed by an inverted microscope (Motic AE31E TRI, 10× eyepieces, 4×, 10×, 20×, 40× objectives) and imaged by a CCD camera (ProgRes CF scan, Jenoptik, 12.5 megapixel) grafted through the microscope’s photo port.

### 2.9 Preparation of Cryogel Scaffolds

Cryogel scaffolds were fabricated by cross-linking thiolated Ch or S-protected thiolated Ch using heparin as a cross-link agent via reactions between their amine groups and carboxylic groups, respectively. First, Ch-SH or Ch-SS was hydrated and dissolved in 1% NaCl to form a 2% m/v solution. Heparin stock solution containing 5% m/v heparin, 1 M EDC, and 1 M sNHS (equal to fivefold molar amount of carboxylic groups on heparin) was prepared 15 min before mixing with modified Chs. All solutions were kept on ice. The mass ratios of modified Ch to heparin were investigated at four levels of 8:1, 8:2, 8:3, and 8:4, respectively; 10, 20, 30, or 40 μL of the heparin stock solution was diluted with water (if necessary) to obtain the final volume of 40 μL. Thereafter, 40 μL of the heparin solution was added and mixed with 200 μL of Ch-SH/Ch-SS solution in the well of 96-well plate. The final concentration of Ch-SH/Ch-SS was 1.67% m/v. Samples were frozen at −22 °C for 24 h and lyophilized for another 24 h. Dry cryogel scaffolds were cut into discs of 1–2 mm height for characterization and testing. To remove excess EDC and sNHS, cryogel scaffolds were soaked in 96% ethanol five times, each time for 1 h, and let to dry in the vacuum chamber at room temperature. Cryogels prepared from Ch-SH and Ch-SS were denoted as SHg and SSg, respectively.

To study the cryogel structure in wet state and cell penetration and proliferation in cryogel scaffolds by confocal laser scanning microscopy (CLSM), cryogels were labeled with Alexa Fluor 488 dye by mixing heparin with 1% of Alexa Fluor 488-labeled heparin. Accordingly, Alexa Fluor 488 Cadaverine (Invitrogen, A30676) was conjugated with heparin via the EDC/sNHS-mediated reaction. In brief, 1 mg of Alexa Fluor 488 Cadaverine was added to 0.75 mL of the solution containing 0.5% m/v heparin, 16.67 mM EDC, and 16.67 mM sNHS. The reaction mixture was protected from light and shaken at 22 °C and 500 rpm overnight (ThermoMixer C, Eppendorf, Germany). Thereafter, the reaction mixture was dialyzed against water (Spectra/Por *M*_w_ cut-off 3.5 kDa) for 8 h and lyophilized to recover Alexa Fluor 488-labeled heparin.

### 2.10 Characterization of Cryogels

#### 2.10.1 Swelling Properties

The diameter and mass of dry cryogel discs were recorded. They were immersed in deionized water at room temperature for 4 h. Afterward, water was replaced by PBS, and samples were left overnight. The diameter and mass of the swollen cryogels were recorded after the removal of excess buffer on the cryogel surface by tissue papers. Mass swelling ratio was calculated by eq 4, while diameter swelling ratio was the ratio of the swollen cryogel diameter to dry cryogel diameter. Triplicate samples for each cryogel formulation were tested.
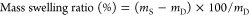
4where *m*_S_ and *m*_D_ are the masses of swollen cryogel and dry cryogel, respectively.

#### 2.10.2 Rheological Properties

The rheological properties of swollen cryogels were evaluated by a rheometer (Haake Mars, ThermoFisher Scientific, Germany) with a rotating-plate (diameter 35 mm) measuring setup. Measurements were carried out in oscillatory amplitude sweep mode at a temperature *T* of 25 °C, frequency *f* of 1 Hz, and shear stress τ in the range 0.1–1000 Pa. Storage modulus *G*’ (Pa) and loss modulus *G*” (Pa) representing the solid-state behavior and liquid-state behavior of the sample, respectively, were recorded. In the logarithmic-scale diagram of *G*’ and *G*” plotted against τ, the linear viscoelastic (LVE) region is the region where the gel returns to its original form when stress is withdrawn, and declining point is the point where elastic deformation is limited and plastic deformation begins (Figure S.6). Declining point is determined as the point where the *G*’ value declines more than 10% from its average value in the LVE region. Beyond this point, the gel network structure begins to break, collapse, or fracture.

#### 2.10.3 Structure of Cryogel in Swollen State

The structure and pore size distribution (PSD) of swollen cryogel were studied by CLSM (Leica TCS SP8, Germany). Appropriate filter sets were used to record z-stacks with 0.5 μm z-step length of the scaffold. Image postprocessing and analysis were performed utilizing the open-source image processing and analysis platforms ImageJ and MatLab. Ilastik, a machine-learning-based segmentation toolkit, was used for segmentation of the scaffold image stacks. To determine the PSD, the segmented scaffold stacks were further analyzed using a custom-written program in MatLab. In short, the regionprops function was utilized to derive the PSD via the estimation of the diameter of circular objects fitting into the pore area within the image slice. The final PSD was determined as a sum of the PSDs of each image slice and displayed as the kernel density estimate.

### 2.11 Cell Penetration and Proliferation in Cryogel Scaffolds

Prior to cell seeding, dry cryogels were sterilized by UV radiation, as described in Section 2.8, for 60 min. Thereafter, they were distributed into a 24-well cell culture plate and immersed in sterilized water for 4 h. The plate was put in the cell incubator. Sterilized water was then replaced by cell culture medium, and the plate was put back in the incubator for 24 h. To aid cell penetration into the gel scaffold, the swollen cryogels were placed on a sterilized filter paper for about 1 min to remove the excess medium from the pore cavities. Gels were put back in the 24-well plate, and 100–150 μL of 3 T3 cell suspension (1 × 10^5^ cells/mL) was slowly dropped onto the top side of the gel block. The gel samples were incubated for 40 min for initial adhesion before 1 mL of culture medium was added to each well. After 2 days, the gel samples were transferred to a new 24-well plate, and cells were cultured, with the medium being replaced every 2 days.

Cell penetration and proliferation in the cryogel scaffolds were visualized and monitored by MTT staining and resazurin assay, respectively. Viable cells can metabolize the yellow MTT to purple formazan crystals accumulated in the cells. On day 10, cell-seeded gels were transferred to wells containing 1 mL of 0.5 mg/mL MTT in RFM and incubated for 3 h. Furthermore, cell proliferation was monitored by resazurin assay.^27^ Cell-seeded cryogels were cultured for 14 days and tested for cell viability on days 2, 5, 7, 10, and 14. On the test day, each cryogel was transferred to a new well and immersed in 500 μL of RFM for 30 min. Afterward, RFM was replaced by 22 μM resazurin solution in RFM, and gels were incubated for 8 h in the cell incubator. Control cryogels without seeding cells were cultured and tested in parallel. Fluorescence intensities were measured using the microplate reader (Tecan Spark, Austria), with the gain value fixed at 70, so that intraday values can be compared. For control, the fluorescence signal from 22 μM resazurin solution as an intraday reference was used. After testing for cell viability, cell-seeded cryogels were transferred back to the old plate and continued culturing.

Cell penetration and proliferation in swollen cryogel scaffolds were further investigated by CLSM. After 5 days of culture, the culture medium was replaced by HBSS modified with 10 mM HEPES, and samples were incubated for 4 h to remove phenol red in the culture medium. The medium was exchanged after the first 2 h. Thereafter, gels were cut into thin slices with a razor blade prior to cell nuclei live staining with Hoechst solution. In brief, the samples were incubated with the staining solution for 5 min and washed two times with HBSS, 5 min each. The gel samples were transferred to an eight-well iBidi μ-slide and observed with the confocal microscope. The raw and segmented image data of the cryogel scaffold, together with the Hoechst-stained nuclei of the cells, are displayed either as maximum projections of the image volume along the *z*- or *y*-direction of the stack or as 3D-rendered image. All fluorescence images were recorded under equal confocal settings.

### 2.12 Statistical Data Analysis

Experiments were carried out with at least three replicate samples. Data are expressed as mean ± SD. Student’s *t* test, assuming unequal variances, was used to analyze the difference between the means of two datasets. One-way analysis of variance (ANOVA) was used to test for differences in the means of three or more datasets. Statistical significance levels: ns, nonsignificant, *p* > 0.1; * *p* < 0.05; ** *p* < 0.01; *** *p* < 0.001.

## 3 Results and Discussion

### 3.1 Synthesis and Characterization of Nac-MDP, Ch-SH, and Ch-SS

The disulfide-bearing ligand NacMDP was successfully synthesized and purified, as confirmed by TLC (Figure S.1), FTIR, and NMR spectra. The ^1^H NMR spectrum of NacMDP is characterized by two singlets that are assigned to the protons of the methyl groups (acetyl CH_3_ at 2.08 ppm and methoxy of the ester at 3.79 ppm). The three signals for the methylene protons of the thiopropanoic acid and the NacME subunits can be found in the range between 2.76 and 3.26 ppm, and the CH proton as a multiplet at 4.89–4.94 ppm. The NH proton appears as a doublet at 6.56 ppm, while the OH proton of the carboxylic group appears as a broad signal between 6.8 and 7.5 ppm (Figure 1B).

FTIR spectrum of NacMDP (Figure 1C) shows three characteristic bands at 1729, 1634, and 1537 cm^–1^ corresponding to the ester C=O, amide I C=O, and amide II C–N stretching vibrations, respectively, inherited from the NacME structure. An additional band at 1707 cm^–1^ that can be assigned to the dimer carboxylic C=O stretching confirmed the successful coupling with the propionic acid moiety. This is further confirmed by the increase in the intensity of bands at 2954, 2916, and 2849 cm^–1^, characterizing the stretching vibrations of the methyl and methylene groups. The broad band at 3305 cm^–1^ can be assigned to the combination of hydrogen-bonded amide N–H and carboxylic O–H groups, while aliphatic disulfide C–S–S–C stretching vibrations give rise to weak bands at the 500 cm^–1^ region.^28^

**Figure 3:**
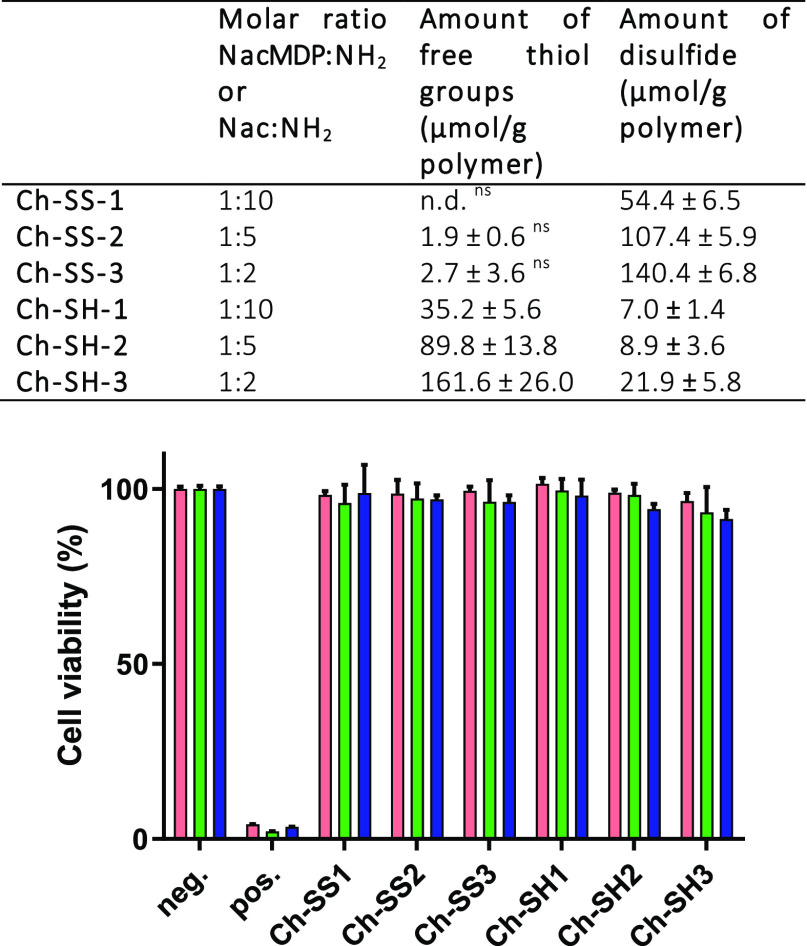
(Top) Amount of free thiol and disulfide in Ch-SH and Ch-SS synthesized at different reaction molar ratios of Nac/NacMDP to the amine groups on chitosan; n.d.: not detected, ns: nonsignificant (one-way ANOVA, *p* = 0.34, considering the amounts of free thiol in Ch-SS-1 samples was 0 μmol/g polymer). (Bottom) Viability of HT29 cells (pink columns), 3T3 cells (green columns), and rat chondrocytes (blue columns) when incubated with modified polymers for 24 h. Polymers were hydrated and swollen in culture medium for 6 h before the whole swollen polymer and medium were transferred to cell layers. Culture medium served as negative control (neg.), while 0.1% Triton X100 served as positive control (pos.).

Ch was grafted with Nac and NacMDP to yield Ch-SH and Ch-SS, respectively. The conjugates of Ch with Nac are well established, but there has been no study on the interaction of these materials with cells.^20,25^ Ch FTIR spectrum is characterized by bands at 1616 and 1511 cm^–1^ corresponding to the amide I C=O (*N*-acetyl) stretching and primary amine N–H (mainly) mixed with amide N–H bending vibrations (Figure 2). Bands at 1061 and 1032 cm^–1^ can be attributed to the C–O stretching and O–H bending vibrations, while the band at 1151 cm^–1^ corresponds to the C–O–C bridge asymmetric stretching vibration. The introduction of Nac or NacMDP to Ch backbones increased the density of secondary amide, leading to the appearance of a band at 1715 cm^–1^ with an increased band intensity as the Nac/NacMDP:NH_2_ molar ratio increased. This band was blue-shifted due to the protonation of amide groups during synthesis.^29^ Along with the appearance of the band at 1715 cm^–1^, there was an increase in intensity and dominance of the band at ∼1560 cm^–1^ (amide N–H bending vibration) over the band at 1511 cm^–1^ (amine N–H bending vibration) (Figure S.2). This can be explained by the decrease of free amine groups and increase of amide groups on Ch. Signals of C–S and S–H vibrations are generally weak, overlapped, and hard to be assigned in the infrared spectra.^28^Figure 3 shows the amount of free thiol and disulfide groups per gram of thiolated Chs or S-protected thiolated Chs synthesized with different molar ratios of Nac/NacMDP:NH_2_. We targeted three theoretical degrees of modification of 50, 100, and 150 μmol thiol or disulfide per gram of polymer. As can be seen, Ch-SS polymers contain negligible amounts of free thiol, while Ch-SH polymers contain considerable amounts of disulfide bonds. This can be explained by the partial oxidation of free thiols on Ch-SHs to form −S–S– linkages with unbound Nac or with free thiols on Ch-SHs per se.

Cytotoxicity of Ch-SH and Ch-SS at different degrees of modification was evaluated on two immortal cell lines: HT29 and 3T3, and primary rat chondrocytes. As illustrated in Figure 3, cell viabilities were in most cases higher than 90% and not significantly different from that of negative control samples, indicating good cytocompatibility of Ch-SHs and Ch-SSs to HT29, 3T3, and rat chondrocytes. There was no correlation between the degree of Ch modification and cytotoxicity. Results were in line with previous studies as Ch-Nac conjugates have been shown to be safe and well tolerated in humans.^30^ Dünnhaupt et al. synthesized S-protected thiolated Chs by coupling 6-mercaptonicotinamide (6-MNA) to Ch-thioglycolic acid (Ch-TGAs) at different degrees of modification. They showed that Ch-TGAs were more cytotoxic than Ch-TGA-MNAs.^31^ It seems that the type of ligand rather than degree of modification is the more decisive factor affecting cell viability.

### 3.2 Cell Adhesion and Proliferation on Chitosan-, Ch-SH-, and Ch-SS-Coated Surfaces

For anchorage-dependent cells, the foremost condition for cell growth, proliferation, and differentiation is the adherence of cells to a solid substrate. Therefore, cell–surface interactions are essential knowledge to develop biomaterials for cell culturing, cell delivery, and TE.^32−34^ In this study, cell adhesion and proliferation on plastic Petri dishes coated with seven polymers, i.e., Ch, Ch-SH-1, Ch-SH-2, Ch-SH-3, Ch-SS-1, Ch-SS-2, and Ch-SS-3 were investigated using three adherent cell types, i.e., HT29, 3 T3, and rat chondrocytes. Films cast by all polymers except Ch were smooth and transparent. The six-well cell culture plate and uncoated plastic Petri dishes served as positive and negative controls, respectively. Results showed that all the three cell lines can attach, grow, and proliferate on the six-well cell culture plate, whereas they cannot attach and grow on the hydrophobic surfaces of uncoated plastic Petri dishes.

**Figure 4:**
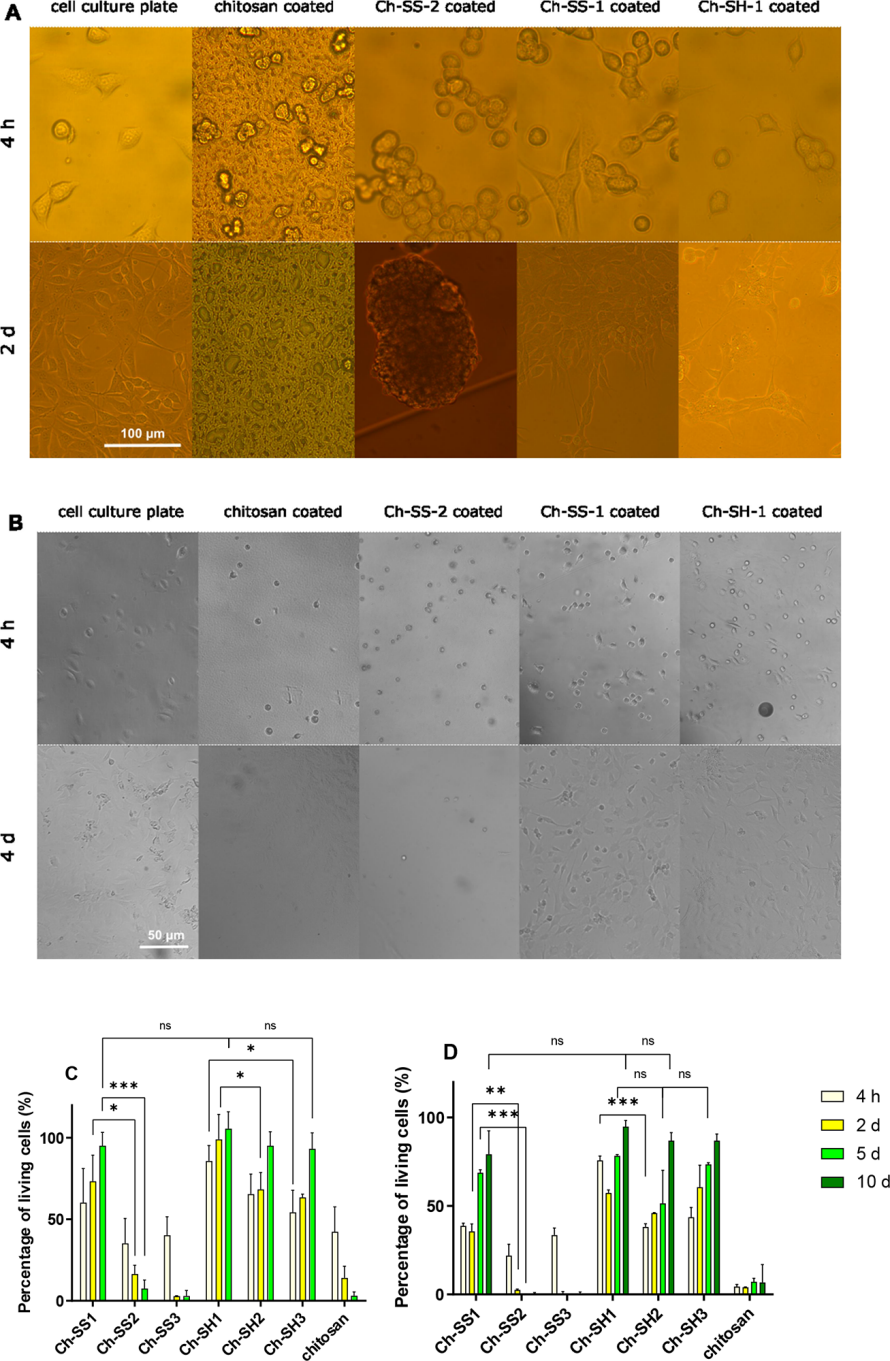
(A) Images of 3T3 cell attachment and growth on Ch-SH-1-coated and Ch-SS-1-coated Petri dishes after 4 h of cell seeding and after culturing for 2 days. Cells were unable to attach and grow on Petri dishes coated with chitosan, Ch-SS-2, and Ch-SS-3 (image not shown). 3T3 cells seeded and cultured on six-well cell culture plates served as controls. Images were taken by a CCD camera (ProgRes CF scan, Jenoptik, 12.5 megapixel) connected to an inverted microscope (Motic AE31E TRI), observed with 10× eyepiece and 20× magnification objective. (B) Images of rat chondrocyte attachment and growth on Ch-SH-1-coated, and Ch-SS-1-coated Petri dishes after 4 h of cell seeding and after culturing for 4 days. Cells were unable to attach and grow on Petri dishes coated with chitosan, Ch-SS-2, and Ch-SS-3 (image not shown). Chondrocytes seeded and cultured on six-well cell culture plate served as controls. Images were taken by a CCD camera (ProgRes CF scan, Jenoptik, 12.5 megapixel) connected to an inverted microscope (Motic AE31E TRI), observed with 10× eyepiece and 10× magnification objective. Attachment and proliferation on different polymer-coated surfaces of 3T3 (C) and rat chondrocyte cells (D). Values are percentage of amount of living cells in a polymer-coated Petri dish compared to that of a six-well cell culture plate (served as 100% viability). Medium was removed 4 h after cell seeding to remove unbound cells. Data are expressed as mean ± SD, *n* = 3. Student’s *t* test assuming unequal variances was used to compare the means between two groups. Statistical significance: ns, nonsignificant *p* > 0.1, * *p* < 0.05, ** *p* < 0.01, *** *p* < 0.001.

HT29 cells seeded on Ch-coated, Ch-SH-coated, and Ch-SS-coated Petri dishes did not attach to dish surfaces and stayed in round shape after 4 h of seeding. After 48 h, cell clusters were formed in uncoated and polymer-coated dishes (Figure S.3). 3 T3 cells and rat chondrocytes could attach and proliferate on Ch-SH-1-, Ch-SH-2-, Ch-SH-3-, and Ch-SS-1-coated Petri dishes, with the best performance on Ch-SH-1-coated dishes (Figure 4). At the same inoculum cell density, 3T3 cells seemed to better attach to polymer-coated surfaces and grow and proliferate faster than chondrocytes. Although 3T3 and chondrocyte cells can attach to surfaces coated with Ch-SS-2, Ch-SS-3, or Ch within 4 h after seeding, they cannot grow and proliferate on these surfaces. As depicted in Figure 4A,B, cells seeded on Ch-SS-1- and Ch-SH-1-coated dishes for 4 h had elongated morphology similar to the cells seeded in the cell culture plate, whereas cells seeded on Ch-, Ch-SS-2-, or Ch-SS-3-coated dishes were still mostly in compact and round shape. After 2 days of incubation, these round-shaped cells agglomerated to form cell clusters or cell spheroids, as shown in Figures 4A and S.4. Cho et al. observed cell spheroid formation on the plate coated with N-hexanoyl glycol Ch.^35^ In the scope of this study, we did not focus on the cell spheroid formation. It could help, however, to maintain the undifferentiated state of pluripotent stem cells in in vitro cell culture, and sometimes that is the requirement for cell sources used in TE.^36^

Cell–substrate adhesion is initiated by the attachment of cells on substrate via nonspecific interaction like electrostatic interaction, followed by specific interactions of transmembrane cell adhesion molecules (CAMs), i.e., integrins, and the substrate via intermediate molecules like serum fibronectin and vitronectin.^37,38^ Ch is a linear biodegradable polysaccharide built by randomly distributed D-glucosamine and *N*-acetyl-D-glucosamine units. It has a similar structure to glycosaminoglycans (GAGs) that are indispensable constituents of proteoglycans, one of the major elements of the ECM. GAGs are strongly negatively charged and are able to bind serum adhesion factors, growth factors, ECM proteins, and CAMs providing cell adhesion capability.^39−41^ Unlike GAGs, Ch is positively charged, and thus its interactions with those factors and receptors are less effective.^42,43^ This explains the poor cell adhesion to Ch substrates, although their positively charged surfaces can favor initial cell–substrate interactions. Similar results were observed by other research groups.^9,10^ Ch-SHs and Ch-SSs were synthesized by the amidation of Ch. Hence, the amount of free amine groups on modified Ch molecules decreased, resulting in less positively charged molecules and thus better interaction with serum adhesion factors. The presence of thiol and disulfide groups on Ch-SHs and Ch-SSs, respectively, provides these molecules the ability to nonspecifically interact with cysteine–/methionine-rich regions of different proteins via thiol–thiol oxidation or thiol–disulfide exchange reactions.^20,44,45^ Therefore, Ch-SSs and Ch-SHs are able to bind to adhesion factors and growth factors in serum in cell culture medium, resulting in more efficient cell adhesion and proliferation on Ch-SH-coated and Ch-SS-1-coated dishes.

Moreover, cell surface thiols––exofacial thiols––can also play an important role in cellular uptake and cell adhesion.^15^ The presence of about 20 nmol of free reactive thiols on the surface of 10^6^ HT1080 cells^14^ suggests that thiol–disulfide exchange reactions may occur between S-protected thiolated polymers and those exofacial thiols at the cell surface promoting the initial cell attachment on thiolated polymers. Although Ch-SS-2 and Ch-SS-3 contained higher amounts of disulfide groups than Ch-SS-1 (Figure 3), coating Petri dishes with these polymers did not result in more pronounced cell adhesion and proliferation but the formation of cell cluster and cell spheroids. Ch-SH-2 and Ch-SH-3 contained higher amounts of thiol groups than Ch-SH-1 but showed even lower cell adhesion within 4 h. After 5 days, there was no significant difference between Ch-SHs in cell adhesion and proliferation (Figure 4C,D). Higher degrees of modification made the Ch-SH and Ch-SS molecules more hydrophobic and thus seemed to lower the cell attachment. Since the ligand NacMDP is bulkier and more hydrophobic than Nac, the surfaces of Ch-SS-2 and Ch-SS-3 films are more hydrophobic than those of Ch-SH-2 and Ch-SH-3, leading to lower cell attachment and proliferation on Ch-SS-2- and Ch-SS-3-coated dishes. It can be concluded that (i) Chs modified with Nac are advantageous over Chs modified with NacMDP regarding cell attachment and proliferation, and (ii) a high degree of modification of Ch (e.g., more than 100 μmol NacMDP per gram of polymer) may result in a modified polymer with higher hydrophobicity, rendering the polymer film less suitable for cell attachment. Ch-SS-1 and Ch-SH-1 were chosen to prepare cryogels in further experiments.

### 3.3 Properties of Cryogel Scaffolds

Cryogel scaffolds have many advantages in TE. They provide robust macroporous 3D structures with high surface area, open pore morphology, and interconnected pore network that can facilitate the supply of nutrients, removal of waste metabolites, and blood vessel ingrowth.^46^ Cryogels are formed via the following steps: phase separation with ice crystal formation, cross-linking, and polymerization, followed by removal of ice crystals to form an interconnected porous cryogel network. As the solvent freezes, two distinct phases are formed: the frozen phase and the unfrozen liquid microphase.^47^ Concurrently, the solute molecules condensed in the unfrozen liquid microphase begin to interact, resulting in the formation of a gel network. After cross-linking has occurred, the cryogel is lyophilized, leading to the formation of an interconnected porous structure with polymer walls surrounding the pores. In this study, heparin was primarily used as a cross-link agent for cryogel formation and thereafter as a binding region of various growth factors that regulate different cellular processes and tissue regeneration.^48,49^

**Figure 5:**
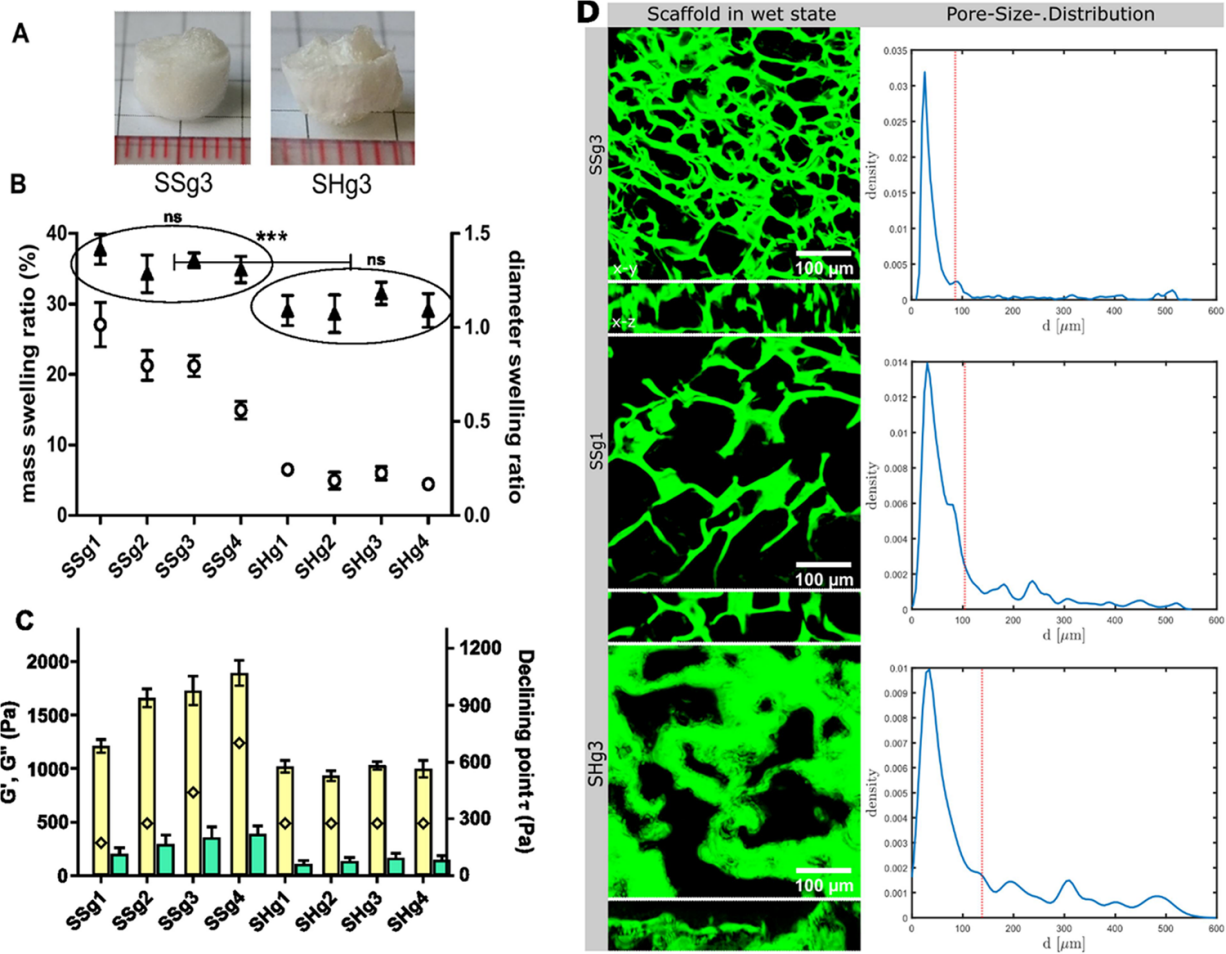
(A) Cryogels SSg3 and SHg3 in dry form. (B) Mass swelling ratios and diameter swelling ratios of cryogels. SSg1, SSg2, SSg3, and SSg4 are cryogels made from Ch-SS-1, and the mass ratios of Ch-SS-1 to heparin are 8:1, 8:2, 8:3 and 8:4, respectively. SHg1, SHg2, SHg3, and SHg4 are cryogels made from Ch-SH-1, with mass ratios of Ch-SH-1 to heparin being 8:1, 8:2, 8:3, and 8:4, respectively. White circles denote mass swelling ratios of cryogels after the removal of fluid on the surface, and black triangles denote the diameter swelling ratios of swollen gels. Values are expressed as mean ± SD, *n* = 3. Diameter swelling ratios of four SSg cryogels (circled data, left) were compared using one-way ANOVA. Similarly, the diameter swelling ratios of four SHg cryogels (circled data, right) were compared using one-way ANOVA. The difference between group 1 “pooled data of all SSg cryogels” and group 2 “pooled data of all SHg cryogels” was analyzed using Student’s *t* test, assuming unequal variances; ns: nonsignificant *p* > 0.1, *** *p* < 0.001. (C) Rheological characters of swollen SHg and SSg cryogels. The numbers 1, 2, 3, or 4 in the cryogel names indicate the mass ratios of thiolated chitosan to heparin of 8:1, 8:2, 8:3, or 8:4, respectively. Yellow columns denote storage modulus *G*’, and blue columns denote loss modulus (*G*”). *G*’ and *G*” measurements were carried out in oscillatory amplitude sweep mode at 25 °C, frequency of 1 Hz, and shear stress τ in the range 0.1–1000 Pa. Diamond denotes the shear stress τ at the declining point where the *G*’ value declines more than 10% from its average value in the linear viscoelastic (LVE) region. (D) Confocal images of Alexa Fluor 488-labeled cryogel scaffolds in wet state in *x*–*y* and *x*–*z* projections (left panel), and pore size distribution (PSD) of SSg3, SSg1, and SHg3; red line indicates mean diameter in μm. PSD is displayed as the kernel density estimate.

Cryogels prepared from Ch-SH-1 and Ch-SS-1 were termed SHg and SSg, respectively, and mass ratios of Ch-SH-1 or Ch-SS-1 to heparin were 8:1, 8:2, 8:3, and 8:4 corresponding to the mixing levels of 1, 2, 3 and 4, respectively. SSg3 with Ch-SS-1-to-heparin mass ratio of 8:3 had a more homogeneous and ordered structure with the mean ± SD pore diameter of 86 ± 61 μm, while SHg3 with the Ch-SH-1-to-heparin mass ratio of 8:3 had the pore size distributed over a wider range, with mean ± SD of 138 ± 140 μm. SSg1 with the Ch-SS-1-to-heparin mass ratio of 8:1 had a larger mean pore size of 103 ± 110 μm (Figure 5D). SSg cryogels having the same diameter as the mold (7 mm) were brittle, while SHg cryogels shrinking to 5–6 mm in diameter were stiffer (Figure 5A). SSg cryogels quickly absorbed water and swelled in less than 1 min, whereas it took several hours for SHg cryogels to fully absorb water and swell. All gels reached swelling equilibrium in water within 4 h. As shown in Figure 5B, higher amounts of heparin in SSg cryogels resulted in lower mass swelling ratios, indicating higher cross-linking degrees in the gel structures. Therefore, tuning the Ch-SS-to-heparin ratio can generate cryogels with the desired structure and pore size that best support the growth and differentiation of different cell types. A similar trend was not clear in the case of SHg cryogels, and mass swelling ratios of SHg cryogels were overall lower than that of SSg cryogels. Besides, the average diameter swelling ratio of SHg cryogels was 1.1 and lower than that of SSg cryogels 1.4 (*p* < 0.01). This was in agreement with the fact that SHg cryogel scaffolds had much thicker walls (Figure 5D) and thus more rigid structures than SSg cryogel scaffolds. Larger pore size and thicker walls of SHg3 might be due to the disulfide bridge formation between the free thiols on Ch-SH-1 molecules, drawing these molecules closer. This was supported by the observation that 2% Ch-SH-1 solution used to prepare cryogel scaffolds had higher dynamic viscosity than 2% Ch-SS-1 solution (141 mPa s vs 96 mPa s, calculated from rheological measurement under the following conditions: amplitude sweep mode at 25 °C, plate–plate setup, gap of 0.5 mm, frequency of 1 Hz, and shear stress of 0.1–100 Pa) and the shrinking shapes of the dry SHg cryogels. Both SSg and SHg swollen cryogels can quickly desorb water when being placed on a filter paper and reabsorb when being placed back in water, indicating the formation of interconnected macropore network in the cryogel scaffold. This is a favorable feature that allows the diffusion of nutrients and wastes in and out of cryogel scaffolds in TE.^46^

Mechanical parameters of TE scaffolds are among the fundamental factors affecting the cell spreading behavior, migration, and proliferation as cells are sensitive to their surrounding microenvironment.^50−53^ Like most soft tissues in the body, scaffolds are viscoelastic, i.e., they have viscous (*G*”) and elastic (*G*’) moduli. While *G*” is less commonly used to evaluate the gel scaffold for TE, substrates with high loss modulus *G*” have been shown to facilitate cell spreading and proliferation.^51^*G*’ is proportional with the level of cross-linking within the gel network and thus is proportional with the gel stiffness, which is a major factor affecting the cell growth and proliferation in cell culture scaffolds.^54,55^ For SSg and SHg cryogels, *G*’ is always greater than *G*”, which means the elastic or solid behavior of the swollen cryogel scaffolds dominates their viscous or liquid behavior. At the same polymer concentration, SSg cryogels showed higher elastic moduli than SHg cryogels. Their *G*’ and shear stress at declining point values also showed proportional increases with heparin amounts (Figure 5C), indicating increases in the gel network strength and cross-link density. The shear stress at the declining point could be used to indicate the point when the gel network begins to collapse. As shown in Figure 5C, SSg4 with the highest amount of heparin has the stiffest gel network. For SHg cryogels, there was no clear correlation between the rheological properties and ratio of heparin. It seemed that thiol–thiol interactions between Ch-SH molecules had restricted the amidation reaction with heparin due to steric effects or had outperformed the amide cross-links induced by heparin in SHg cryogels.

### 3.4 Cell Penetration and Proliferation in Cryogel Scaffolds

**Figure 6:**
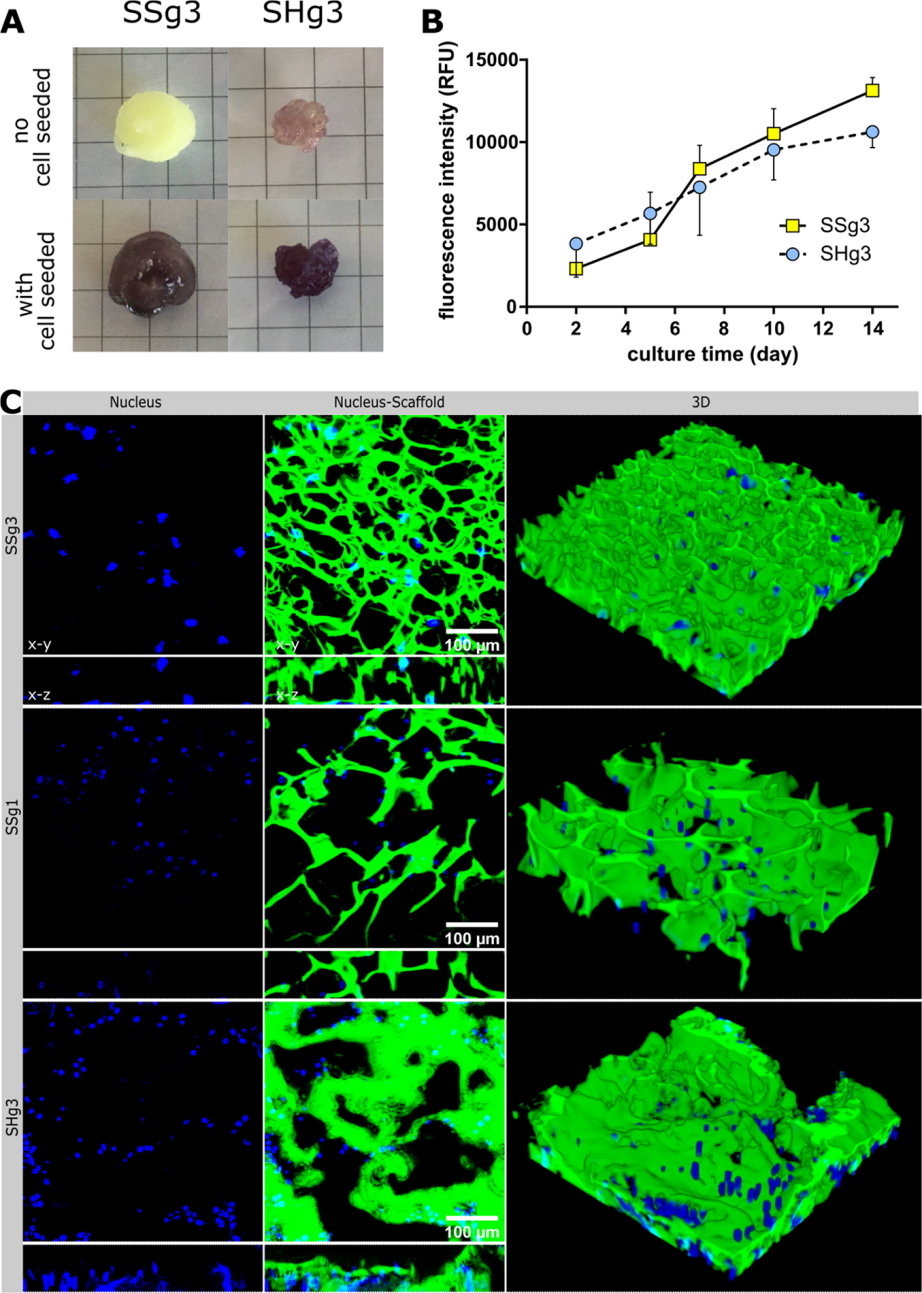
(A) SSg3 and SHg3 cryogels without and with 3T3 cell seeding and culturing for 10 days visualized by the MTT reagent. Purple color indicates the presence of live cells. In the case of SHg3 cryogel, thiol moieties in the gel scaffold reduce the MTT reagent, generating a light purple color (upper right corner photo), irrespective of the presence of cells or the cell viability. (B) 3T3 cells growth on SSg3 and SHg3 cryogel scaffolds; cell proliferation was monitored by resazurin assay. (C) From left to right: *x*–*y* and *x*–*z* projections showing nucleus-only and nucleus in cryogel scaffold, and 3D confocal microimages of 3T3 cell attachment and growth in cryogel scaffolds (SHg3, SSg1, and SSg3) at day 5 of culture. Green: cryogel scaffolds labeled with Alexa Fluor 488; blue dots/clusters are cell nuclei stained with Hoechst dye.

The potential of SHg and SSg cryogels as scaffolds for TE was investigated by evaluating the penetration and proliferation of 3T3 fibroblast cells seeded onto swollen cryogel scaffolds. To visualize the cell proliferation, cryogel scaffolds were incubated with the MTT reagent 10 days after cell seeding. Live cells will metabolize and convert soluble MTT to purple formazan crystals accumulating in cytoplasmic granules. Cell-seeded SHg3 and SSg3 scaffolds turned to purple color, indicating cell adhesion and growth in the scaffolds (Figure 6A). SHg3 cryogel without seeded cell showed light purple color, as free thiols in Ch-SH can chemically reduce MTT to formazan depositing in the gel network. In this aspect, resazurin assay is far less interfered by functional groups like thiols, amines, or carboxylic acids.^56^Figure 6B shows cell proliferation in SSg3 and SHg3 over time monitored by resazurin. CLSM images gave a closer look at the cells located in the 3D structure of the cryogel scaffold. In line with the results of MTT staining scaffolds, 3T3 cells were shown to migrate into the interconnected macropores inside the scaffold, attach to pore walls, and proliferate throughout the cryogel scaffold after 5 days in culture (Figure 6C).

## 4 Conclusions

New S-protected thiolated Chs were synthesized by conjugating the disulfide-bearing ligand 3-((2-acetamido-3-methoxy-3-oxopropyl)dithio) propanoic acid to the Ch backbone. Three S-protected thiolated Chs: Ch-SS-1, Ch-SS-2, and Ch-SS-3 with low, medium, and high degree of modification, respectively, were successfully prepared. They showed good cytocompatibility and neglectable cytotoxicity. Ch-SS-1 is a good substrate for cell attachment, growth, and proliferation, whereas Ch-SS-2 and Ch-SS-3 showed poor cell adhesion properties because of the increased hydrophobicity of polymer membranes as the degree of modification increased. Interestingly, cells cultured on Ch-SS-2 and Ch-SS-3 can form cell spheroids that are desired to maintain the undifferentiated state of stem cells. On the other hand, all three Nac-conjugated Chs, Ch-SH-1, Ch-SH-2, and Ch-SH-3, were shown to be good substrates for cell adhesion, growth, and proliferation. The higher degree of modification of these polymers did not significantly reduce their cell adhesion and proliferation. Thiolated Chs and S-protected thiolated Chs can promote cell adhesion and proliferation via nonspecific thiol–thiol or thiol/disulfide exchange interactions with exofacial thiols on the cell surface and thiol-containing regions in adhesion protein structures.

For 3D cell culture, Ch-SS polymers are superior to Ch-SH polymers. Cryogel scaffolds from Ch-SS-1 and Ch-SH-1 were fabricated using heparin as a cross-linking agent. SSg cryogels structured by Ch-SS-1 are homogeneous scaffolds with tunable pore size and mechanical properties when changing the mass ratio between Ch-SS-1 and heparin. Differently, Ch-SH-1 forms cryogel scaffolds with thick walls and nontunable mechanical properties. A reason for this behavior is that thiol–thiol interactions between Ch-SH-1 molecules might have outperformed and limited the cross-link reactions of Ch-SH-1 with heparin molecules. SSg cryogel scaffolds with their interconnected microporous structure showed good cell migration, adhesion, and proliferation. Therefore, the newly synthesized Ch-SS-1 can be a potential material for TE and regenerative medicine.

## Supporting Information Available

Isolation and culture of rat chondrocytes; TLC and FTIR spectra; cell images; and rheological properties (PDF)
