# Supplementary material for: S-Protected Thiolated Chitosan versus Thiolated Chitosan as Cell Adhesive Biomaterials for Tissue Engineering
Source: ACS Appl Mater Interfaces. 2023 Aug 18;15(34):40304–16. doi: 10.1021/acsami.3c09337 (PMC10472333; doi:10.1021/acsami.3c09337)
Supplement: Supplementary file 1 — am3c09337_si_001.pdf [file am3c09337_si_001.pdf]

S-PROTECTED THIOLATED CHITOSAN VS. THIOLATED CHITOSAN AS CELL ADHESIVE BIOMATERIALS FOR TISSUE ENGINEERING

Bao Le-Vinh[a,b], Christian Steinbring[a], Nguyet-Minh Nguyen Le[a,b], Barbara Matuszczak[c], Andreas Bernkop-Schnürch[a*]

[a] Department of Pharmaceutical Technology, Institute of Pharmacy, University of Innsbruck, Innrain 80/82, 6020 Innsbruck, Austria

[b] Department of Industrial Pharmacy, Faculty of Pharmacy, University of Medicine and Pharmacy at Ho Chi Minh city, 700000 Ho Chi Minh city, Vietnam

[c] Department of Pharmaceutical Chemistry, Institute of Pharmacy, University of Innsbruck, Innrain 80-82, 6020, Innsbruck, Austria

*Corresponding author:
Department of Pharmaceutical Technology,
Institute of Pharmacy, University of Innsbruck
Innrain 80/82, 6020 Innsbruck, Austria
Tel.: +43-512-507 58601 30. Fax: +43-512-507 58699
E-mail: andreas.bernkop@uibk.ac.at

1. Isolation and culture of rat chondrocytes

Rat chondrocytes were isolated according to previously described methods [1] [2]. An in vivo study was approved by the Ethical Committee of Austria (Vienna) and performed following the Principles of Laboratory Animal Care. Male Sprague−Dawley rats (250−300 g) were obtained from Janvier Labs (Saint Berthevin, France).

Materials: 5x 100 mm petri dishes (overnight in LAF with UV on), PBS containing Penicillin-streptomycin (P/S) (ratio: 1 mL of P/S solution + 100 mL of PBS), 0.5 mg/mL collagenase D in DMEM.

Procedure:

- Remove the skin and soft tissues from the hind leg using scissors and pincers.

- Dislocate the femurs and discard the soft tissues.

- Isolate the femoral head, condyles, and tibial plateau; place in a 50 mL tube filled with PBS.

- Rinse with PBS, put into a 100 mm petri dish, the cartilages are chopped finely (1 mm cube) using a blade.

- The tissue is then incubated in DMEM containing 0.5 mg/mL collagenase D at 37 °C, 5% CO_2 in the cell incubator for the matrix to be digested.

- After overnight incubation, isolated chondrocytes are cultured in DMEM supplemented with 10% fetal bovine serum (Gibco) and 1% penicillin, in a T75 flask (bottom area 75 cm^2) and sub-cultured when confluency reached 80%. The morphology of the cells is confirmed via microscopy on day 2.

- When the cells have been cultured from passage 0 to 1, they are preserved via freezing in liquid nitrogen, and thawed before further experimentation.

2. TLC and FTIR spectra

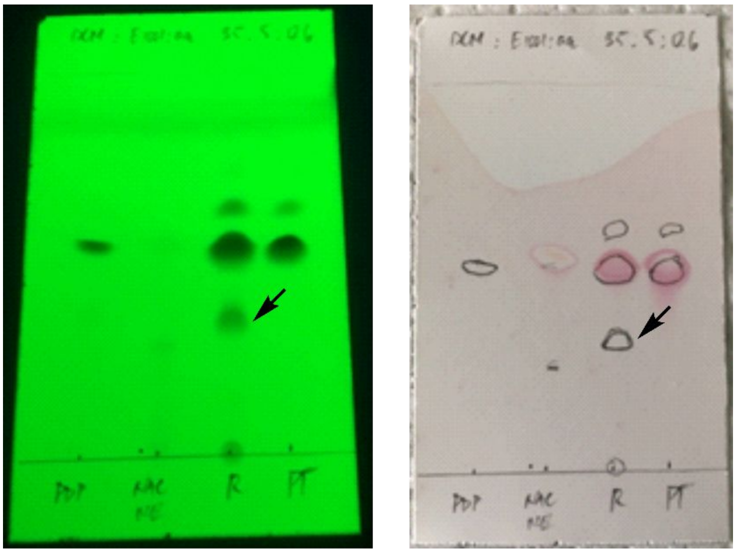

Figure S. 1. TLC plates under UV 254 nm (left) and after being sprayed with ninhydrin solution (right). Mobile phase: DCM:Ethanol:acetic acid 95:5:0.6. Abbreviations on TLC plates: PDP: 3-(2-pyridyldithio) propionic acid, NACME: N-acetyl L-cysteine methyl ester, R: reaction mixture, PT: pyridine-2-thiol. The product 3-((2-acetamido-3-methoxy-3-oxopropyl)dithio) propanoic acid (NacMDP) spot is indicated by the black arrow. Compounds containing –SH and $–NH_2$ such as PT and NacME react with ninhydrin to produce purple compounds, while PDP and NacMDP containing –S-S- do not react with ninhydrin.

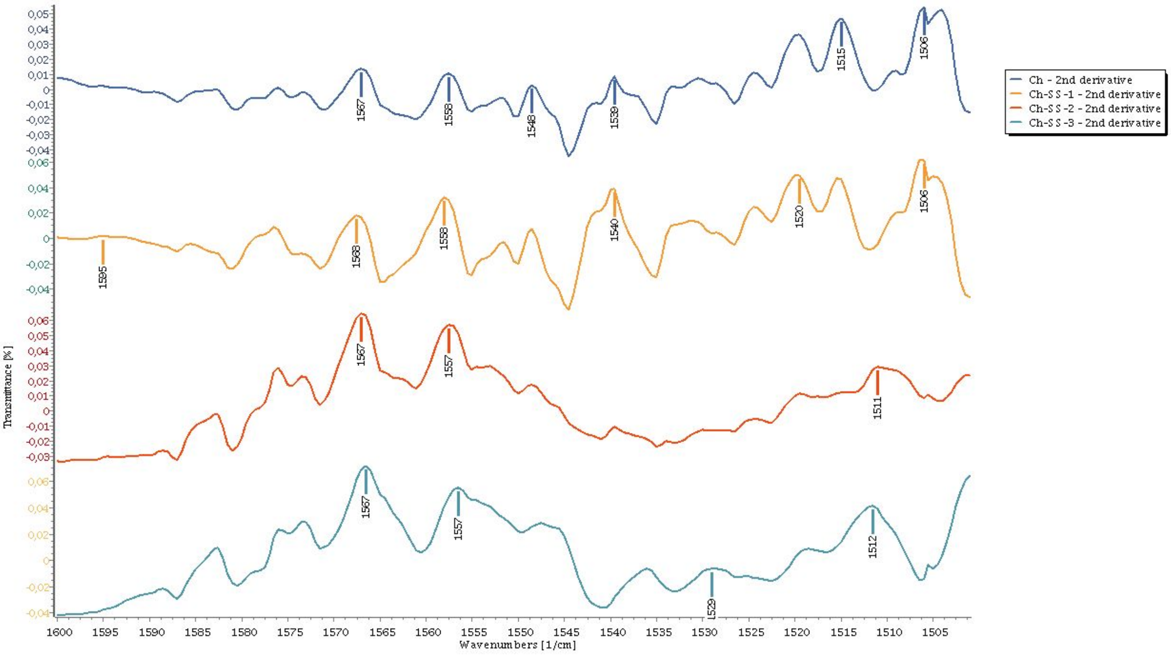

Figure S. 2. Second derivative FTIR spectra from 1600–1500 cm^{-1} of chitosan (Ch), and S-protected thiolated chitosans Ch-SS-1, Ch-SS-2, and Ch-SS-3 corresponding to NacMDP:NH_2 molar ratios of 1:10, 1:5, and 1:2, respectively. As molar ratio increases, intensity ratio of band at ~1568/1560 cm^{-1} to band at 1506/1511 cm^{-1} increases. Bands at 1511 cm^{-1} correspond to amide I C=O (N-acetyl) stretching and primary amine N-H bending vibrations, and bands at ~1560 cm^{-1} correspond to amide N-H bending vibration. This intensity ratio increases and peak shift (1506 -> 1511 cm^{-1}) indicates the decrease of free amine groups and increase of amide groups on chitosan molecules due to the success of amidation reaction.

3. Cell images

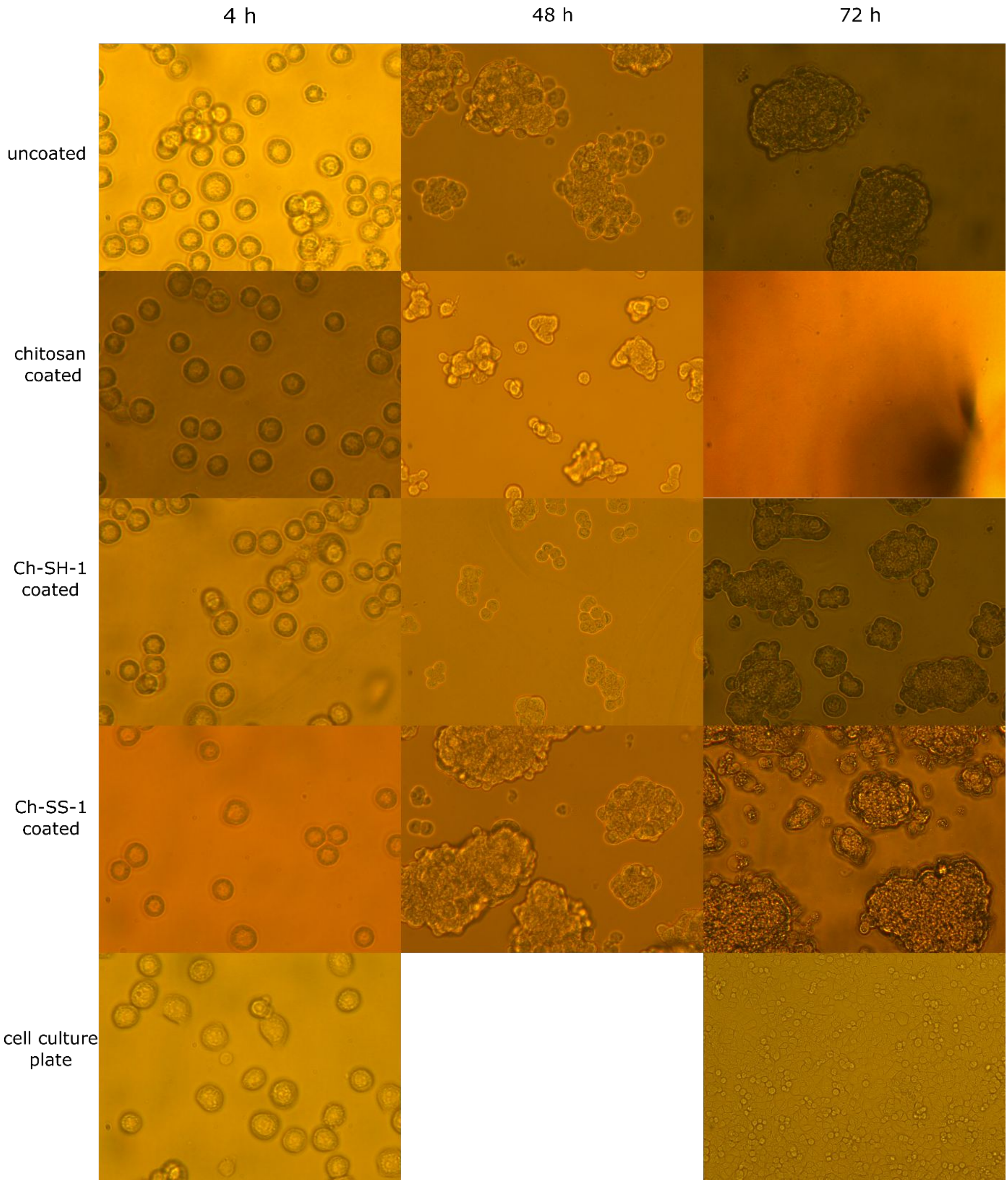

Figure S. 3. Representative images of HT29 cell attachment and growth on uncoated, chitosan-coated, Ch-SH-1-coated, and Ch-SS-1-coated petri dishes after 4 h of cell seeding, and after culturing for 48 h and 72 h. Cells seeded and cultured on 6-well cell culture plate served as controls. Images were taken by a CCD camera (ProgRes CF scan, Jenoptik, 12.5 megapixel) connected to an inverted microscope

(Motic AE31E TRI), observed with 10x eyepiece and 20x magnification objective. On cell culture plate, HT29 cells change their morphology after 4 h of seeding and reach 100% confluency after 72 h of culture (image taken with 4x objective). HT29 cells seeded on uncoated, chitosan-coated, Ch-SH-1-coated, and Ch-SS-1-coated petri dishes do not attach to the surface, stay in round shape after 4h of seeding, and tend to form cell clusters after 48 h.

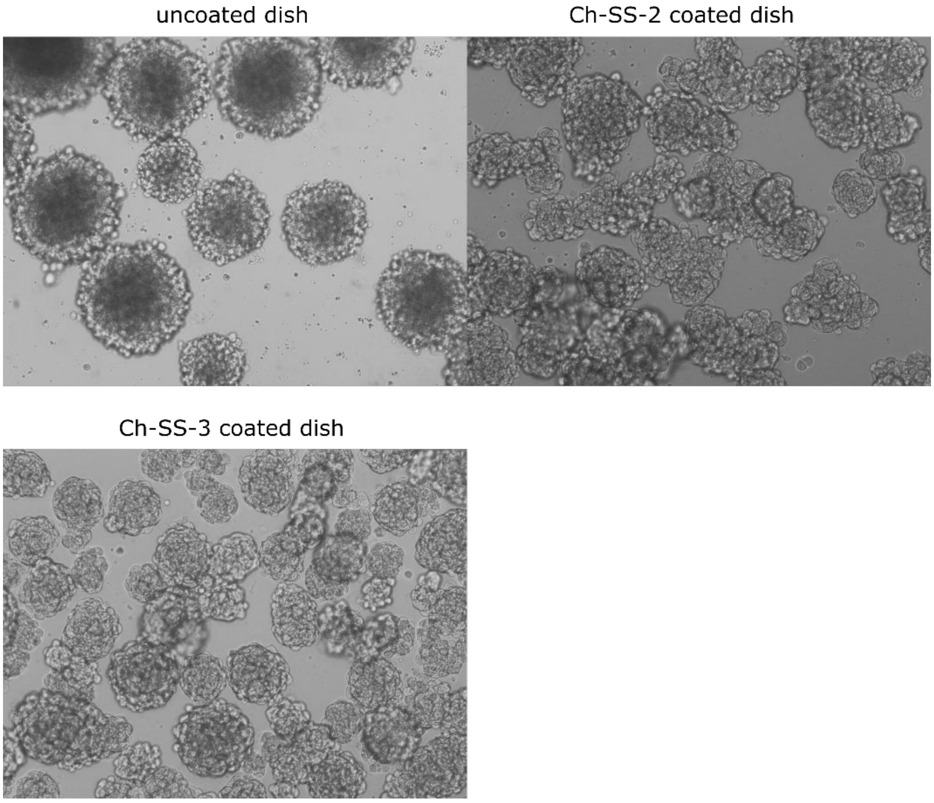

Figure S. 4. 3T3 cell spheroids or clusters on uncoated, Ch-SS-2 coated, and Ch-SS-3 coated petri dishes after 2 days of incubation. Images were taken by a CCD camera (ProgRes CF scan, Jenoptik, 12.5 megapixel) connected to an inverted microscope (Motic AE31E TRI), observed with 10x eyepiece and 20x magnification objective.

4. Rheological properties

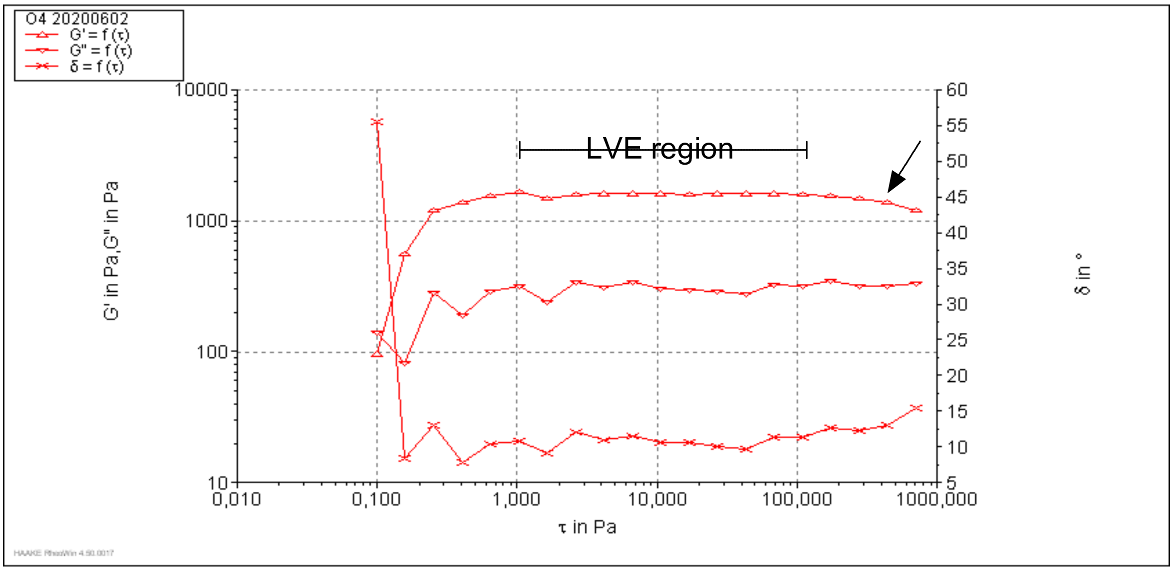

Figure S. 5. Logarithmic diagram of G', G'' versus shear stress τ with depicted linear viscoelastic (LVE) region and declining point (arrow)

Reference

[1] M. Gosset, F. Berenbaum, S. Thirion, C. Jacques, Primary culture and phenotyping of murine chondrocytes, Nat. Protoc. 3 (2008) 1253–1260. https://doi.org/10.1038/nprot.2008.95.

[2] M.O. Cho, Z. Li, H.-E. Shim, I.-S. Cho, M. Nurunnabi, H. Park, K.Y. Lee, S.-H. Moon, K.-S. Kim, S.-W. Kang, K.M. Huh, Bioinspired tuning of glycol chitosan for 3D cell culture, NPG Asia Mater. 8 (2016) e309–e309. https://doi.org/10.1038/am.2016.130.
